# Association of MMP-9 polymorphisms with diabetic nephropathy risk: A protocol for systematic review and meta-analysis

**DOI:** 10.1097/MD.0000000000022278

**Published:** 2020-09-18

**Authors:** Yan Xie, Zhixue Wang, Lin Chang, Guotao Chen

**Affiliations:** a Health Management Centre, The First Affiliated Hospital of Army Medical University; b Department of Clinical Laboratory; c Department of Nephrology, Bishan Hospital, Bishan District, Chongqing, Chongqing, China.

**Keywords:** diabetic nephropathy, meta-analysis, MMP-9, polymorphism

## Abstract

**Background:**

Diabetic nephropathy (DN) is a multifactorial disease with gene–environment interaction resulting in progressive renal function damage. Multiple studies have assessed the association between matrix metalloproteinase-9 (MMP-9) gene promoter polymorphism and DN susceptibility. However, the results are inconclusive. In the present study, we will conduct a meta-analysis to further examine this relationship more precisely.

**Methods:**

Electronic databases (Pubmed, Web of Science, Embase, Google Scholar, Wanfang, China Biological Medicine and China National Knowledge Infrastructure) will be used to search clinical case–control studies about MMP-9 polymorphism and DN published until 18 August 2020. The language will be restricted to Chinese and English. Two reviewers will take charge of completing the selection of study, the extraction of data as well as the assessment of study quality independently. The Newcastle-Ottawa Scale will be used to evaluate the study quality. We will evaluate the association under 5 genetic models. Fixed-effects or random-effects models will be used to calculate the effect sizes of odds ratio and 95% confidence intervals. Afterwards, subgroup analysis will be conducted in terms of the ethnicity and genotyping method. Additionally, sensitivity analysis will be performed via sequentially omitting each of the included studies one at a time. The funnel plots, Egger regression test, and Begg rank correlation test will be used to test the potential publication bias. All the statistical analyses will be performed using Review Manager 5.3 and Stata 12.0.

**Results:**

This protocol reported according to the Preferred Reporting ltems for Systematic Reviews and Meta-Analyses Protocols (PRISMA-P) statement. This study will provide a better understanding of the association between MMP-9 polymorphisms and DN risk.

**Conclusion:**

Publishing this protocol will minimize the potential bias related to data mining, thus contributing to generation of reliable evidence.

**OSF registration number:**

DOI 10.17605/OSF.IO/H5FS4

## 1 Introduction

Diabetes mellitus (DM) is approaching epidemic proportions and has become a major public health problem worldwide. According to the latest report, nearly 463 million people suffer from diabetes, and the population is expected to reach 700 million by 2045.^[1]^ Unfortunately, the increasing prevalence of diabetic nephropathy (DN) is consistent with the striking rise in the prevalence of diabetes.^[2,3]^ DN is a the most serious microvascular complication that progresses gradually in about 30% to 40% of individuals with DM.^[4,5]^ The main pathological changes of DN are diverse, such as the accumulation of growth factors, advanced glycation end products, changes in hormones and hemodynamics, resulting in persistent proteinuria, constant decreased kidney function, and hypertension.^[5]^ Kidney disease caused by diabetes is a major factor contributing to the global burden of disease. At present, DN is not only the primary cause of chronic kidney disease worldwide, but also the main cause of end-stage renal disease (ESRD) requiring renal replacement therapy such as dialysis or transplantation.^[6,7]^ The incident cases of chronic kidney disease due to T2DM worldwide in 2017 had increased by 74% compared to 1990; total disability-adjusted life years had increased by 113%.^[8]^ Most notably, most of the excess risk of cardiovascular disease and all-cause mortality for patients with diabetes is associated with the existence of DN.^[9]^ Every year, management of DN is related to huge long-term health care costs in most countries.^[10]^

Although the pathogenesis of DN is still unclear, in recent years, a growing body of evidence has indicated to inherited factors together with environmental factors playing a role in the development of DN.^[11,12]^ In DM-DN, genetic susceptibility is mainly characterized by familial aggregation, and the prevalence of DN is different among different ethnic groups.^[13,14]^ Recently, due to the development of genetic methods, multiple single nucleotide polymorphisms (SNPs) associated with DN susceptibility have been discovered, such as SLC12A3,^[15]^ ACACβ,^[16]^ AGTR1,^[16]^ ELMO1,^[17,18]^ MTHFR,^[19]^ SLC12A3,^[20]^ CNDP1, and CNDP2.^[21]^ The matrix metallopeptidase-9 (MMP-9) gene is also one of the widely studied SNPs in DN. The occurrence of DN is closely associated with inflammation, and matrix metalloproteinases (MMP) plays important roles in the development of DN.^[22]^ The MMP family members participate in the breakdown of extracellular matrix in physiological processes, such as embryonic development, tissue remodeling, and reproduction, whereas MMP-9 is the 9th member of them. MMP-9 may play an essential role in leukocyte migration and in local proteolysis of the extracellular matrix. In addition, several studies showed that MMP-9 is a momentous inflammatory marker involved in the pathophysiological process of DN.^[23]^ The expression and activity of MMP-9 in DN may be regulated by a variety of mechanisms, and SNPs in the *MMP-9* gene may affect its expression. The *MMP-9* gene is located in chromosome 20q13.12. The variations in the regulatory region of *MMP-9* gene have been proved to be an essential factor affecting the expression of MMP-9.^[24]^

A series of epidemiological researches have assessed the association between the *MMP-9* gene polymorphism and the risk of DN.^[25–27]^ The most common variants are microsatellites (CA)n^[28]^ and rs3918242 (-1562C/T)^[26,27]^ promoter polymorphisms. A recent meta-analysis provided potential evidence of the association between MMP-9 gene promoter polymorphism and the risk of diabetic microvascular complications,^[26]^ but there is no system review specifically evaluating MMP-9 SNPs in DN to date. Fortunately, meta-analysis has been applied successfully in multiple researches to annihilate confounding results and evaluate the relationship based on a larger sample size. Therefore, we will perform a systematic review and meta-analysis of the existing literature to provide a more precise and comprehensive examination of the association between MMP-9 polymorphism and DN risk.

## 2 Methods/design

### 2.1 Study registration

The protocol has been registered in the Open Science Framework (OSF) (registration number: DOI 10.17605/OSF.IO/H5FS4). This systematic review and meta-analysis will be reported in conformity with the preferred reporting items for systematic reviews and meta-analysis protocols (PRISMA-P) 2015.^[29]^ The review does not involve the assessment of patients’ individual information or rights, thus there is no need to obtain approval from an ethical institution.

### 2.2 Inclusion criteria

Studies will be included in this meta-analysis based on the following criteria:

1. Types of studies: all case–control studies associated with the susceptibility of MMP-9 polymorphisms to DN will be incorporated in our review. No restriction will be put on the publication date or status of the study.
2. Types of participants: participants suffered from DN will be included in the meta-analysis. Control subjects should be defined as diabetes individuals without DN or healthy individuals. No restrictions will be placed on age, sex, or country.
3. Data of the MMP-9 polymorphism could be available on genotype distributions for estimating the odds ratio (OR) with its 95% confidence interval (CI), or adequate data are provided to estimate the corresponding estimate effect (OR, 95% CI).
4. Outcome: DN risk comparsions.

### 2.3 Exclusion criteria

Studies will be excluded from the meta-analysis according to the following criteria: conference abstracts, case reports, unpublished articles, review paper, in vitro or animal study, family-based studies, study has insufficient data for genotyping distribution calculation, repeat reports (for researches using the same sample in different publications, only the most recent or comprehensive information will be included following careful identification).

### 2.4 Search strategy

Electronic searching will focus on databases of Pubmed, Web of Science, Embase, Google Scholar, Wanfang, China Biological Medicine, and China National Knowledge Infrastructure (CNKI) databases, with the temporal from the inception of database to 18 August 2020. A combination of Medical Subject Headings (MeSH) alongside free terms will be used to hunt all the potentially eligible publications. The language will be restricted to English and Chinese. The following terms will be used (“SNP” or “mutation” or “genetic polymorphism” or “variation” or “polymorphism” or “single nucleotide polymorphism” or “variant”) and (“diabetic Nephropathies" or “diabetic Nephropathy” or “DN” or “Diabetic Kidney Disease” or “DKD”) and (“matrix metalloproteinase 9” or “MMP9”). We will also supplement this search by manually searching the reference lists of related articles.

### 2.5 Data collection and analysis

#### 2.5.1 Selection of studies

**Figure 1:**
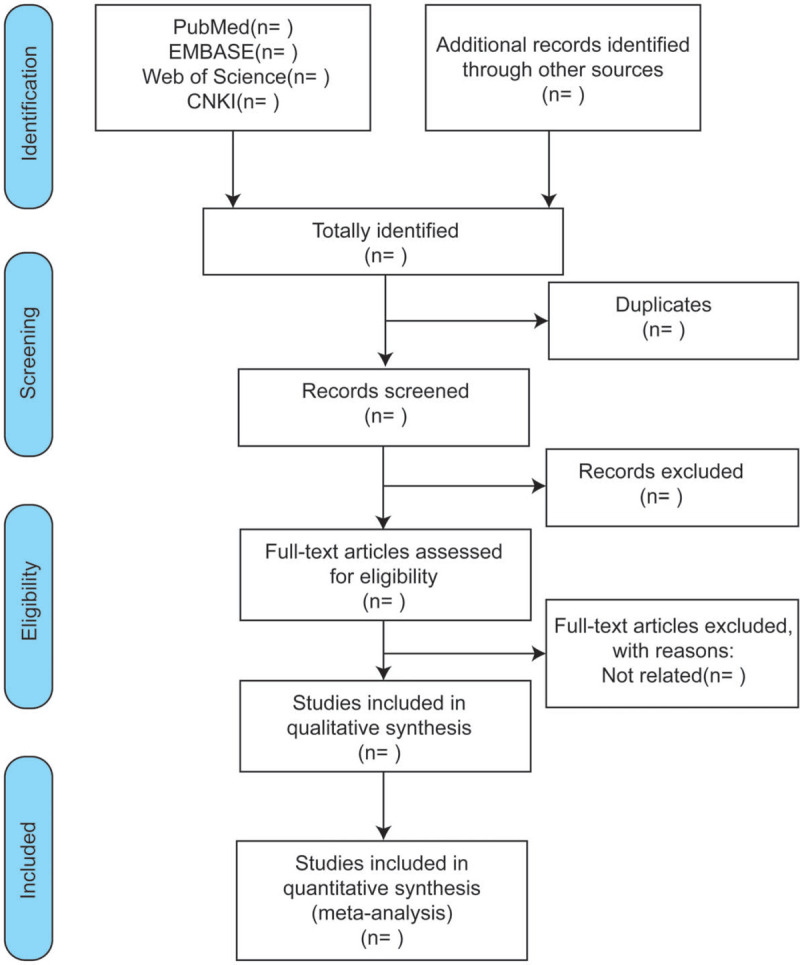
Flow chart of study selection.

The article screening process will involve reading the title first, followed by the abstract and full text to determine whether the study should be included. Members of our team will be professionally trained on the purpose and process of the review beforehand. Two reviewers (YX and ZW) will perform the selection process independently, with cases of disagreement resolved consulting a third reviewer (GC). A flowchart of information pertaining to identification, screening, eligibility, and final datasets selected will be constructed according to PRISMA guidelines,^[29]^ as is shown in Figure 1.

#### 2.5.2 Data extraction

Data from each eligible studies will be extracted, including surname of the first author, year of publication, country of origin, ethnicity of each study population, numbers of cases and controls, sex, mean age, genotyping methods, genotype distribution, and allele frequencies in case/control groups for the MMP-9 polymorphisms. We will also examine the Hardy-Weinberg equilibrium (HWE) of genotype distributions in the control group.

#### 2.5.3 Study quality assessment

The quality of all the included studies will be evaluated by 2 reviewers (YX and LC) independently according to the Newcastle-Ottawa scale (NOS), which is used to evaluate the quality of observational studies.^[30]^ Disagreement will be reported and resolved by a third reviewer (GC). Three broad perspectives of each study quality will be scored: the selection of the study groups, the comparability of the case and control groups, determination of the exposure or outcome of interest in the studies. The NOS values arrange from 0 to 9. Studies with a score of ≥6 are considered to be of high quality.

#### 2.5.4 Dealing with missing data

The reason for the loss of data missed in the period of data screening and extraction will be identified here. We will attempt to contact the authors if the data of potential studies are insufficient, missing, or vague. These studies will be excluded only if the data are not available through the method described above.

#### 2.5.5 Statistical analysis

The *χ*^2^ test will be used to evaluate the offset of frequencies of MMP-9 polymorphisms from the expected values under the HWE among healthy controls. If *P* < .05, the study is considered not consistent with HWE. The strength of the association between DN and *MMP-9* gene mutation will be evaluated the pooled ORs with their corresponding 95% CIs. We will evaluate the association under five genetic models: allelic genetic model (a vs A), the dominant genetic model (aa + Aa versus AA), the recessive genetic model (aa vs Aa + AA), the homozygote model (aa vs AA), and the heterozygote genetic model (Aa versus AA). (“a" and “A" represent the mutant allele and the wild-type allele, respectively). Then the most plausible genetic model of inherence will be determined according to the relationships between the 5 pairwise comparisons. After that the underlying genetic model is confirmed, the counts of genotypes will be collapsed into 2 categories to obtain the merged results. The heterogeneity among studies will be assessed utilizing a *χ*^2^ test-based Q statistic and *I*^2^. If *P*_Q__statistic_ > .05 or *I*^2^ < 50%, the fixed-effected model will be merged; otherwise, the random-effected model will be selected. The significance of the pooled ORs will be determined by *Z*-test, with *P* < .05 considered statistically significant. All statistical analyses will be performed by Review Manager 5.3 (Cochrane Collaboration, Oxford, UK) and Stata version 12.0 (Stata Corporation, College Station, TX).

#### 2.5.6 Assessment of heterogeneity

Heterogeneity among the included studies will be evaluated by *I*^2^ statistic. A fixed-effects or random-effects model will be utilized to measure pooled OR in the absence or presence of heterogeneity, respectively. When a set of studies exhibit an obvious heterogeneity, factors leading to the heterogeneity will be discussed, such as the characteristics of patients and the variation degree in exposure. Subgroup analysis, meta regression analysis, and sensitivity analysis will be conducted to explore potential sources of heterogeneity across studies when statistical heterogeneity is detected.

#### 2.5.7 Subgroup analysis

We will carry out subgroup analyses of the relationships between MMP-9 genetic polymorphisms and the risk of DN, according to clinical subtype of DM, different ethnicity, and genotyping method, and so on.

#### 2.5.8 Sensitivity analysis

We will also perform sensitivity analysis to measure the robustness and reliability of the pooled results by sequentially omitting each of the included studies one at a time.

#### 2.5.9 Assessment of publication biases

Egger regression test and Begg rank correlation test will be used to test the potential publication bias. The level of *P* < .05 will be considered significant. In addition, the funnel plots will also be used to examine the publication bias if there are >10 eligible studies.

#### 2.5.10 Grading the evidence quality

We will utilize GRADE method to evaluate the evidence quality of the results obtained.^[31]^ The evaluation involves risk of bias exhibited by studies, the heterogeneity between groups, estimate precision of effect, evidence directness, and publication risk of bias. The evidence quality will be classified into 4 grades: high quality, moderate quality, low quality, and very low quality.

## 3 Discussion

With the dramatic increase in DM incidence, the numbers of people with DN and end-stage renal disease are also increasing continuously.^[32]^ Unfortunately, although glycemic control measures can reduce the proportion of diabetic patients progressing to DN to a certain extent, these approaches cannot avoid the risk of DN.^[6,33]^ DN can lead to both premature death and high mortality, and is also associated with huge medical expenses around the world. Early prevention and intervention strategies are very crucial to reduce the occurrence of kidney disease in diabetic patients. Identifying the genetic components of DN is the critical area in diabetes research because clarifying the genes related to DN will affect all efforts toward understanding of the molecular and mechanism level, its cure, and prevention. Moreover, among the genetic factors involved, the discovery of SNPs in the genes related to DN also has a major impact on disease prognosis. Therefore, studies on polymorphisms of these genes (including MMP-9) are conducted to identify high-risk patients and design targeted therapeutic strategies to prevent severe complications in patients’ later future.

At present, although there are many studies on the relationship between MMP- 9 polymorphisms and DN risk. There is no systematic evaluation on the cumulative evidence that proves the association. We will undertake a systematic review and meta-analysis to clarify the association between SNPs of MMP-9 and susceptibility for DN. The advantages of our study will be: the most recent literature will be included; as to the exploration of heterogeneity, post hoc subgroup analysis will be avoided as much as possible; to improve the credibility of the results, we will perform the sensitivity analysis of each genetic model. Consequently, publishing this protocol will reduce potential biases related to data mining, thus contributing to generation of reliable evidence.

## Author contributions

**Conceptualization:** Yan Xie.

**Investigation:** Zhixue Wang.

**Supervision:** Guotao Chen.

**Writing – original draft:** Lin Chang.

**Writing – review & editing:** Yan Xie, Zhixue Wang.

## Abbreviations

Abbreviations: CI = confidence interval, DM = diabetes mellitus, DN = diabetic nephropathy, ESRD = end-stage renal disease, HWE = Hardy-Weinberg equilibrium, MMP-9 = matrix metalloproteinase-9, NOS = Newcastle–Ottawa Scale, OR = odds ratio, PRISMA-P = Systematic Reviews and Meta-Analyses Protocols, SNP = single-nucleotide polymorphism.
